# Effects of alfalfa levels on carcass traits, meat quality, fatty acid composition, amino acid profile, and gut microflora composition of Heigai pigs

**DOI:** 10.3389/fnut.2022.975455

**Published:** 2022-09-30

**Authors:** Jie Li, Shu Zhang, Xin Gu, Jintang Xie, Xiaodong Zhu, Yizhen Wang, Tizhong Shan

**Affiliations:** ^1^Institute of Feed Science, College of Animal Sciences, Zhejiang Univeristy, Hangzhou, China; ^2^The Key Laboratory of Molecular Animal Nutrition, Zhejiang University, Ministry of Education, Hangzhou, Zhejiang, China; ^3^Key Laboratory of Animal Feed and Nutrition of Zhejiang Province, Hangzhou, Zhejiang, China; ^4^Shandong Chunteng Food Co. Ltd., Zaozhuang, Shandong, China

**Keywords:** alfalfa meal, dietary fiber, Heigai pig, meat quality, growth performance, gut microbiota

## Abstract

Recent years have witnessed a dramatic increase in the demand for healthy and high-quality pork. Alfalfa, one of the most popular perennial forages, is considered a rich source of highly nutritional forage for livestock feed, as it contains over 90% insoluble dietary fiber. Nevertheless, there is a paucity of data confirming the effects of adding alfalfa on pork quality, amino acid composition, and intestinal microbiota composition. Therefore, the objective of this study was to investigate the effects of different dietary levels of alfalfa on carcass traits, meat quality, amino acid and fatty acid composition, and the intestinal microbiota of Heigai pigs. A total of 72 finishing Heigai pigs were randomly assigned to two groups (*n* = 36), with six replicate groups and six pigs per replication. The two experimental diets were formulated to include graded levels of alfalfa, 20% (AM20) and 30% (AM30). The results showed that adding 30% alfalfa meal did not affect the growth performance of Heigai pigs but significantly reduced backfat thickness (*P* < 0.05), pH (*P* < 0.05), increased the a^*^ value, b^*^ value, and flavor amino acid and essential amino acid contents in longissimus dorsi muscle (LDM). In addition, AM30 didn't affect colonic microbiota abundance but significantly reduced the relative abundances of three phyla, such as *Verrucomicrobia*, and 43 genera, such as *Akkermansia*, and significantly increased the relative abundances of 47 genera, such as *Prevotella-2*. Overall, these results advocate for a diet containing 30% alfalfa to improve meat quality by changing the intestinal microflora composition without affecting the growth performance of Heigai pigs, which provides compelling evidence for the use of alfalfa to relieve the pressure on corn and soybean meal demand and produce high-quality pork.

## Introduction

With improvements in living standards, there is an increasing demand for high-quality and good tasting livestock products (1). Meat quality is a vital economic trait in the meat industry, with freshness and nutritional value being the key factors that govern consumers' acceptance. However, excessive pursuit of a greater growth rate and lean percentage has led to a deterioration in meat quality. In addition, as the shortage of feed resources becomes increasingly prominent, using different kinds of byproducts rich in fiber as candidates for feed additives has become the focus of intense study in recent years. As one of the most commonly studied prebiotics, which are generally fermented by the gut microbiota, dietary fiber is supposed to provide the energy required for microbial fermentation and metabolic end products (2). Grain and food processing byproducts with a high fiber content are increasingly used in pig diet formulations. As the price of pork continues to decline, it is of great significance to explore the higher tolerance of dietary crude fiber for finishing pigs to reduce production costs and alleviate the competition between humans and animals for grain. The addition of appropriate fiber to the diet improves the diversity and metabolic capacity of the distal intestinal microorganisms in finishing pigs but does not change fiber digestibility or growth rate (3), which is consistent with the findings that indicated that a high fiber diet also increased *Coprococcus* eutectic in the distal colon of suckling piglets (4).

Alfalfa, a kind of green fodder in traditional-style pig feeding systems in China, contains mainly insoluble dietary fiber, including cellulose, xylans, and lignin (5, 6). However, other studies have reported that diets with a high fiber content may negatively affect feed intake and nutrient digestibility in growing pigs. To date, there are still relatively few studies on the effects of diets containing different fiber sources and levels on meat quality, fatty acid composition, and the intestinal microflora of Chinese local pig breeds.

The Heigai pig is a local Chinese fatty breed with the specific characteristics of prolificacy, roughage-tolerance, tender meat, strong resistance to diseases, and strong adaptability to the environment. Our previous study found that the dietary polyunsaturated fatty acid ratio and rearing conditions can affect the growth performance and meat quality of Heigai pigs (7, 8), but the effects of fiber levels on Heigai pigs have not been reported. Benefiting from the roughage-tolerance of Heigai pigs, the addition of 20% alfalfa to the diet has become a lively practice for feed cost control without significantly compromising production performance. Along with the increasing demand for high-quality pork, the effect of alfalfa on the gut microbial community of pigs rapidly drew more attention. Therefore, the objective of the present study was to evaluate the effects of varying levels of alfalfa meal on growth performance, carcass traits, meat quality, fatty acid composition, amino acid composition, and microbiota composition in Heigai pigs and to further analyze the effects on microbial community composition, which may be a constructive solution for the development of high-quality pork and the reduction of food competition between humans and animals.

## Materials and methods

### Experimental design, diets, and sample collection

All procedures and housing were approved by the Zhejiang University Animal Care and Use Committee. A total of 72 Heigai finishing boars [body weight (BW): 77.79 ± 6.97 kg] were randomly divided into one of two groups (*n* = 36) administered different levels of alfalfa meal, 20% (AM20) and 30% (AM30). Each treatment group consisted of six replicates, with six pigs per replication. The composition estimated nutritional value and determination analysis of the diets are summarized in Supplementary Table S1. The feed intake per replication was measured every day to calculate the average daily feed intake (ADFI). Average daily gain (ADG) and the ratio of ADFI to ADG (F/G) were calculated using BW measurements that were recorded individually at the beginning of the experiment and before slaughter.

After 68 days of the corresponding feeding, one pig was randomly selected for each replicate. After fasting for 12 h with free access to water, the pigs were then slaughtered humanely. The longissimus dorsi muscle (LDM) on the left half of each carcass between the 3^rd^ to 11^th^ ribs were sampled for further assessment of meat quality. Approximately 150 g of LDM was rapidly collected, immediately frozen in liquid nitrogen, and subsequently stored at −80°C for fatty acid composition amino acid profiles and gene expression analysis. A block of muscle tissue was removed from the body (sized within 0.25 cm^2^) and was placed into a fixative solution (10% formalin) for hematoxylin-eosin staining. Additionally, approximately 4 g of colonic contents was transferred to sterile tubes and immediately frozen at −80°C for 16S rRNA gene amplicon sequencing.

### Measurement of carcass traits and meat quality

Slaughter weight, carcass weight, dressing percentage, backfat thickness, loin muscle area, and lean percentage were measured after slaughter. Backfat thicknesses (BFT) was measured at the shoulder (BFT1), 6^th^ and 7^th^ ribs (BFT2), last rib (BFT3) using a sliding caliper. After that, the average backfat thicknesses (ABFT, average of BFT1, BFT2, and BFT3) were calculated.

The pH value of the LDM muscle between the 4^th^ and 5^th^ ribs were measured at 45 min and 24 h post slaughter using a pH meter (PH-STAR, MATTHAUS, Germany) by direct insertion into the samples. The electrode was calibrated with pH 4.00 and pH 7.00 buffer before determination. Each sample was measured three times, and the average value was used for further analyses. The drip loss was determined by suspending muscle samples standardized for surface area in plastic bags at 4°C for 24 h. Drip loss was expressed as a percentage of the initial weight (9). Marbling scores and meat color scores were scored at 45 min after slaughter according to reference standards (NPPC-1994, America, USA). Meat color was measured at 45 min and 24 h after slaughter at the surface of a 2-cm-thick boneless loin chop using a Minolta CM-2002 (Osaka, Japan) spectrophotometer with CIE lab color system: L^*^ (lightness), a^*^ (red-green), and b^*^ (yellow-blue). The intramuscular fat (IMF) content in the LDM at the thoracolumbar junction was measured by Soxhlet extraction with solvent (anhydrous ether) and expressed as a weight percentage of wet muscle tissue (10). The contents of inosinic acids extracted with 0.5 M perchloric acid stored at 4°C for 2 days after slaughter and then was ground in liquid nitrogen, detected using high-performance liquid chromatography (HPLC) and calculated according to the peak areas of the sample fluid and the standard liquid.

### Fatty acid composition and amino acid profile analysis

Approximately 10 g of LDM tissue was ground into small pieces and treated with 2:1 chloroform-methanol solution to obtain about 2 mg of extracted lipids, which were then redissolved in 2 ml of n-hexane and 1 ml of KOH (0.4 M) for saponification and methylation. Then, total fat was converted into fatty acid methyl esters (FAMEs) and their contents was determined by gas chromatography with a capillary column. GC Chem Station software was used to separate FAMEs. The FAME profiles of the samples were compared with FAME standards to identify the fatty acids in the LDM. Fatty acid content was expressed as a percentage of total fatty acids. Approximately 0.1 g of freeze-dried muscle was ground and hydrolyzed in 10 ml of 6 mol/L hydrochloric acid solution at 110°C for 24 h. The solution was diluted with water to 100 and 1 ml of the supernatant was used for analysis (11). The samples were filtered through a 0.45-μm membrane before analysis (12) with an ion-exchange AA analyzer (L8800, Hitachi, Tokyo, Japan).

### Total RNA extraction and quantitative real-time PCR

Total RNA was extracted from muscle samples using TRIzol reagent (Thermo Fisher Scientific Inc., Waltham, MA, US) according to the manufacturer's instructions. The concentration and integrity of the RNA samples were determined using a NanoDrop 2000 instrument (Gene Company Limited, HK, China) with an OD260:OD280 ratio ranging from 1.8 to 2.0. The reverse transcription system was performed using random primers and a RevertAid First Strand cDNA Synthesis Kit (Thermo Fisher) according to the instructions. The primers for myosin heavy chain (MyHC) I, IIa, IIx, and IIb were described in Han et al. (13), while primers for PFKM, PKM, HK2, and GAPDH were designed using Primer 5.0 software (Premier Biosoft International, Palo Alto, CA). The 2-ΔΔCt method was used to analyze the relative changes in gene expression after normalization to that of GAPDH as the internal control (Supplementary Table S2).

### Hematoxylin-eosin staining

Longissimus dorsi muscle tissue was extracted from the fixative, paraffin-embedded, and sectioned. Paraffin sections were stained using a hematoxylin-eosin staining kit (Servicebio, Wuhan, China). The slides were examined and photographed using an Olympus BX61 fluorescence microscope (Japan). The cell morphological structures were observed.

### Measurement of triglyceride and cholesterol contents

The contents of triglyceride (TG) and total cholesterol (TCHO) in the LDM were determined using commercial kits (TG, A110-1-1; TCHO, A111-1-1) purchased from Nanjing Jiancheng Institute of Bioengineering according to the instructions.

### DNA extraction and sequencing analysis

Total DNA was extracted from intestinal content samples using the E.Z.N.A.^®^Stool DNA Kit (D4015, Omega, Inc., USA) according to the manufacturer's instructions. High-throughput sequencing of 16S rDNA was performed using Illumina HiSeq PE250 (Illumina, San Diego, CA, USA). The V3–V4 hypervariable regions of the bacterial 16S rRNA gene were amplified using forward 341F (5′-CCTACGGGNGGCWGCAG-3′) and reverse (5′-GACTACHVGGGTATCTAATCC-3′). Raw data were subjected to quality control using UPARSE. Clustering of eligible reads was performed using the concept of amplicon sequence variants (ASVs) to generate operational classification units (OTUs) with 97% similarity using Usearch (14).

Alpha diversity and beta diversity were calculated by random normalization to the same sequences. Then, according to the SILVA (release 132) classifier, feature abundance was normalized using the relative abundance of each sample. Beta diversity was calculated by QIIME2, and principal coordinates analysis (PCoA), heatmap analysis, Bray-Curtis similarity clustering, and species abundance analysis were performed using R version 3.4.4.

### Statistical analysis

All data are presented as the mean ± SEM. The differences in various indicators among groups were performed by unpaired two-tailed Student's *t*-tests or one-way ANOVA procedure when appropriate using SPSS 20.0. The results of mRNA expression were analyzed using GraphPad Prism, version 6.0. To compare differences in microbiota composition, the relative colonic abundances at the phylum and genus levels were processed by a non-parametric Mann-Whitney U test with a corrected *P*-value. The differences were considered significant when *P* < 0.05 and a trend was noted when the *P*-value was between 0.05 and 0.10.

## Results

### Growth performance and carcass traits of finishing pigs

The growth performances of Heigai pigs fed diets with two different levels of alfalfa are presented in Table 1. The total weight gain, ADG, and F:G ratio were not affected by dietary treatments, and no interaction between dietary fiber levels and carcass traits including carcass yield, BFT1, BFT2, and lean percentage, were observed (*P* > 0.10). However, BFT3 and ABFT tended to decrease (*P* < 0.05) in pigs offered the AM30 diet compared with pigs offered the AM20 diet (Table 2).

**Table 1:** Effects of different levels of alfalfa meal on growth performance of Heigai pigs.

**Traits^2^**	**Diets^1^**	
	**AM20**	**AM30**
Initial BW (kg)	77.64 ± 6.52	77.93 ± 7.93
Final BW (kg)	111.21 ± 11.52	113.93 ± 10.14
Total weight gain (kg)	33.57 ± 7.84	36.00 ± 3.06
ADG (g/d)	460 ± 120	480 ± 100
F:G ratio	5.75 ± 1.41	5.31 ± 1.57

Values are mean ± SEM (*n* = 10).

1 AM20, 20% alfalfa meal diet; AM30, 30% alfalfa meal diet.

2 BW, body weight; ADG, average daily gain; F:G ratio, ratio of feed to gain.

**Table 2:** Effects of different levels of alfalfa meal on carcass traits of Heigai pigs.

**Traits^2^**	**Diets^1^**	
	**AM20**	**AM30**
Carcass weight (kg)	80.29 ± 11.60	83.09 ± 8.59
Dressing percentage (%)	73.13 ± 2.28	72.52 ± 1.35
BFT1 (mm)	45.36 ± 6.21	40.19 ± 4.40
BFT2 (mm)	32.72 ± 6.40	29.72 ± 4.87
BFT3 (mm)	24.82 ± 5.61^a^	15.91 ± 6.68^b^
ABFT (mm)	34.30 ± 3.76^a^	28.61 ± 3.82^b^

a, b Means in rows with different letters are significantly different (P < 0.05). Values are mean ± SEM (*n* = 6). Dressing percentage (%) = Carcass weight/Slaughter weight × 100%. Lean percentage (%) = Lean weight/Carcass weight × 100%.

1 AM 20, 20% alfalfa meal diet; AM 30, 30% alfalfa meal diet.

2 BFT1, back fat thickness at the shoulder; BFT2, back fat thickness between the 6^*th*^ and 7^*th*^ ribs; BFT3, back fat thickness at last rib; ABFT, average backfat thickness, average (BFT1 + BFT2 + BFT3).

### Meat quality

As shown in Table 3, feeding AM30 significantly increased the a^*^ value, b^*^ value (*P* < 0.05), and a^*^ value_24h_ (*P* < 0.01), but there was no effect on other meat colors. In addition, the pH_45min_ (*P* < 0.01) and ΔpH (*P* < 0.01) of fresh pork were slightly decreased in pigs fed the 30% alfalfa diet compared with pigs fed the 20% alfalfa diet. Other meat quality indicators, including IMF, inosinic acid, marbling score, and drip loss, were not altered by dietary fiber levels (*P* > 0.10).

**Table 3:** Effects of different levels of alfalfa meal on the meat quality of Heigai pigs.

**Meat quality traits^2^**	**Diets^1^**	
	**AM20**	**AM30**
IMF (g/100 g)	3.57 ± 0.48	3.74 ± 0.80
Inosinic acid (mg/kg)	1730.90 ± 97.12	1640.17 ± 230.01
Marbling score	1.57 ± 0.53	2.00 ± 0.58
Drip loss (%)	1.87 ± 0.49	1.96 ± 0.55
pH_45min_	6.60 ± 0.20^A^	5.97 ± 0.26^B^
pH_24h_	5.66 ± 0.07	5.67 ± 0.13
ΔpH	0.94 ± 0.18^A^	0.32 ± 0.18^B^
L* value	35.34 ± 3.47	37.76 ± 1.69
a* value	11.67 ± 0.31^b^	12.78 ± 1.14^a^
b* value	6.95 ± 1.04^b^	8.14 ± 0.66^a^
L* value_24h_	46.41 ± 2.13	45.73 ± 2.06
a* value_24h_	9.28 ± 1.16^B^	16.39 ± 2.36^A^
b* value_24h_	13.27 ± 1.48	13.81 ± 1.90

a, b Means in rows with different letters are significantly different (P < 0.05).

A, B Means in rows with different letters are significantly different (P < 0.01). Values are mean ± SEM (*n* = 6).

1 AM 20, 20% alfalfa meal diet; AM 30, 30% alfalfa meal diet.

2 IMF, intramuscular fat content; ΔpH = pH_45*min*_ – pH_24*h*_.

### Fatty acid profile and amino acid composition in the LDM

Compared with AM20, after feeding AM30 there was no significant effect on the fatty acid composition of the LDM of Heigai pigs with the exception of a significant increase in C18:1 (*P* < 0.05) (Supplementary Table S3). We measured the difference in amino acid composition in the LDM of the pigs fed the different diets. As shown in Table 4, feeding AM30 significantly increased the contents of threonine (*P* < 0.05), serine (*P* < 0.05), glutamate (*P* < 0.05), methionine (*P* < 0.01), leucine (*P* < 0.01), tyrosine (*P* < 0.01), lysine (*P* < 0.01), aspartate (*P* < 0.05), but significantly reduced that of arginine (*P* < 0.01). Moreover, the contents of FAAs, EAAs, and the total amino acids in AM30 were significantly higher than those in AM20.

**Table 4:** Amino acid composition in LDM of Heigai pigs.

**Amino acid (g/100 g)^2^**	**Diets^1^**	
	**AM20**	**AM30**
Arginine	1.52 ± 0.06^A^	0.53 ± 0.09^B^
Threonine	1.23 ± 0.19^b^	1.55 ± 0.23^a^
Serine	0.92 ± 0.11^b^	1.08 ± 0.13^a^
Glutamate	2.71 ± 0.52^b^	3.54 ± 0.51^a^
Proline	0.30 ± 0.28	0.22 ± 0.25
Glycine	0.91 ± 0.13	0.85 ± 0.11
Alanine	1.25 ± 0.18	1.3 ± 0.19
Cysteine	0.13 ± 0.05	0.1 ± 0.04
Valine	0.59 ± 0.5	0.63 ± 0.27
Methionine	0.67 ± 0.05^b^	1.08 ± 0.18^a^
Isoleucine	1.00 ± 0.14	0.92 ± 0.11
Leucine	1.48 ± 0.12^b^	1.85 ± 0.24^a^
Tyrosine	0.75 ± 0.07^b^	2.15 ± 0.28^a^
Phenylalanine	0.91 ± 0.06	0.99 ± 0.12
Lysine	1.15 ± 0.23^b^	1.67 ± 0.13^a^
Histidine	1.58 ± 0.34	1.34 ± 0.22
Aspartate	1.97 ± 0.36^b^	2.89 ± 0.76^a^
FAA	14.46 ± 1.59^b^	16.57 ± 1.87^a^
EAA	9.09 ± 0.92^b^	10.35 ± 1.1^a^
Total	19.07 ± 1.56^b^	22.83 ± 2.48^a^

a, b Means in rows with different letters are significantly different (P < 0.05). Values are mean ± standard error (*n* = 6).

1 AM 20 = 20% alfalfa meal diet; AM 30 = 30% alfalfa meal diet.

2 FAA, flavor amino acids, FAA = ∑(Serine, Glutamate, Proline, Glycine, Alanine, Valine, Methionine, Isoleucine, Leucine, Lysine, Arginine, Aspartate); EAA, essential amino acids, EAA = ∑(Threonine, Valine, Methionine, Isoleucine, Leucine, Phenylalanine, Lysine).

### The relative mRNA levels of genes related to glycolysis and myofiber in the LDM of Heigai pigs

**Figure 1:**
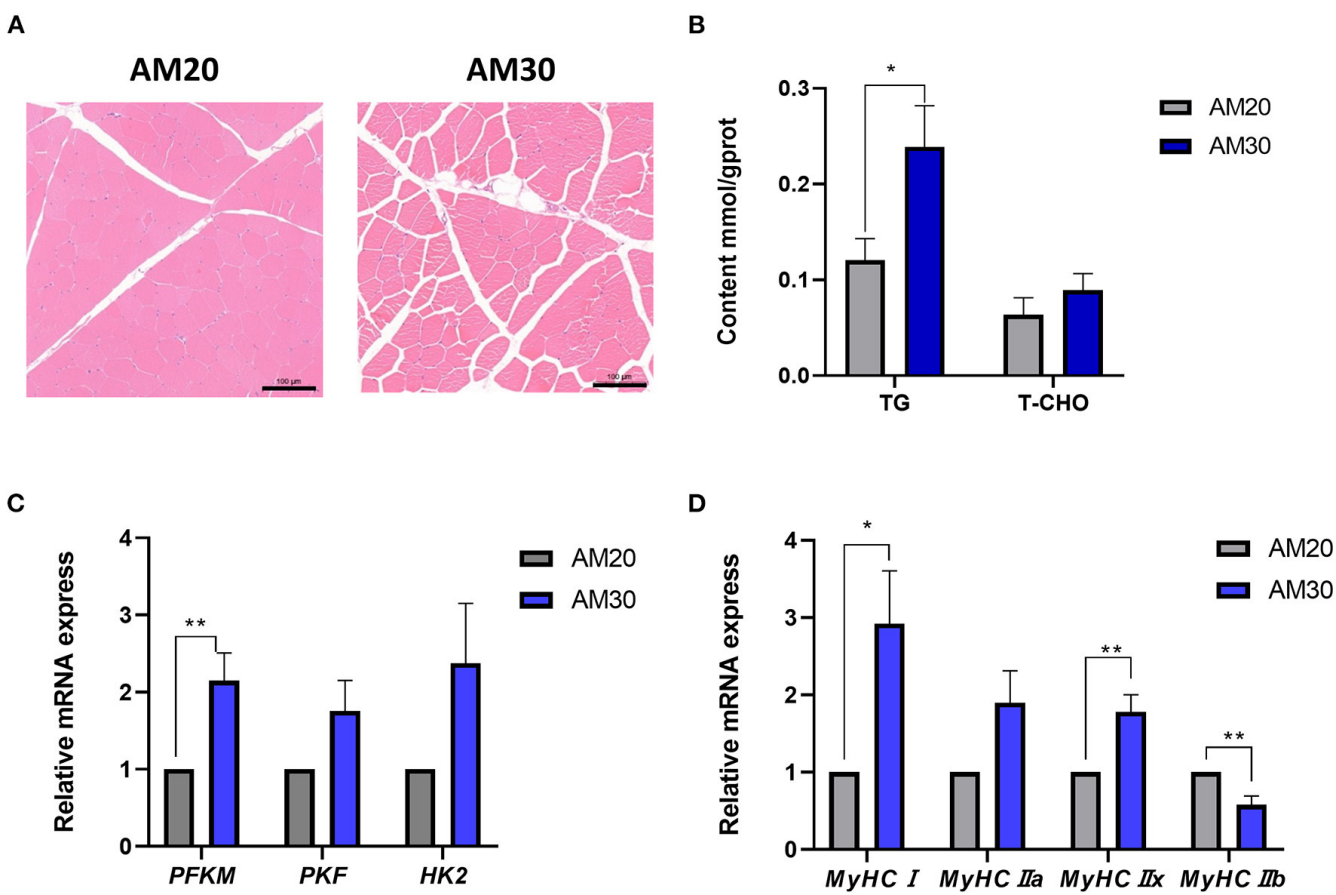
Morphological structure, the contents of triglyceride (TG), total cholesterol (TCHO), and relative mRNA levels of genes related to glycolysis myofiber in LDM of Heigai pigs. **(A)** Histological images (10x) of Hematoxylin-Eosin staining. Scale bar = 100 μm. **(B)** The TG and TCHO contents. **(C)** Relative mRNA expression of genes related to glycolysis (*PFKM, PKF, HK2*). **(D)** Relative mRNA expression of myosin heavy-chain (*MyHC*) isoform genes (*MyHCI, MyHCIIa, MyHCIIb*, and *MyHCIIx*). The mRNA expression was normalized to *GAPDH* gene expression. The data is shown as the mean ± standard error (*n* = 6). *P < 0.05; **P < 0.01.

The histological hematoxylin-eosin staining images showed more IMF deposits in the LDM of the AM30 group with a significantly increased TG content (Figures 1A,B) (*P* < 0.05). It has been widely noted that glycolysis plays an important physiological role in affecting the decline of muscle pH_45min_. To further investigate the mechanism underlying the regulation of pH by different levels of fiber, the expression of genes related to glycolysis was determined, including *PFKM, PKM*, and *HK2*. As shown in Figure 1C, AM30 significantly increased the expression of *PFKM* in the LDM muscle (*P* < 0.05). The same variation trend was observed for the expression of *PKF* and *HK2* without significant differences.

The types of skeletal muscle fibers are critical factors that are closely related to meat quality traits in livestock, especially pH and meat color (15); therefore, the mRNA expression of *MyHC* isoforms was further examined to determine the impact of myofiber types on pH, a^*^ value, and b^*^ value in response to different levels of alfalfa meal. As shown in Figure 1D, dietary supplements with a higher proportion of alfalfa meal significantly increased the gene expression levels of *MyHC?* and *MyHCIIx*, and decreased the expression level of *MyHCIIb* (*P* < 0.05).

### Gut microbiota analysis

**Figure 2:**
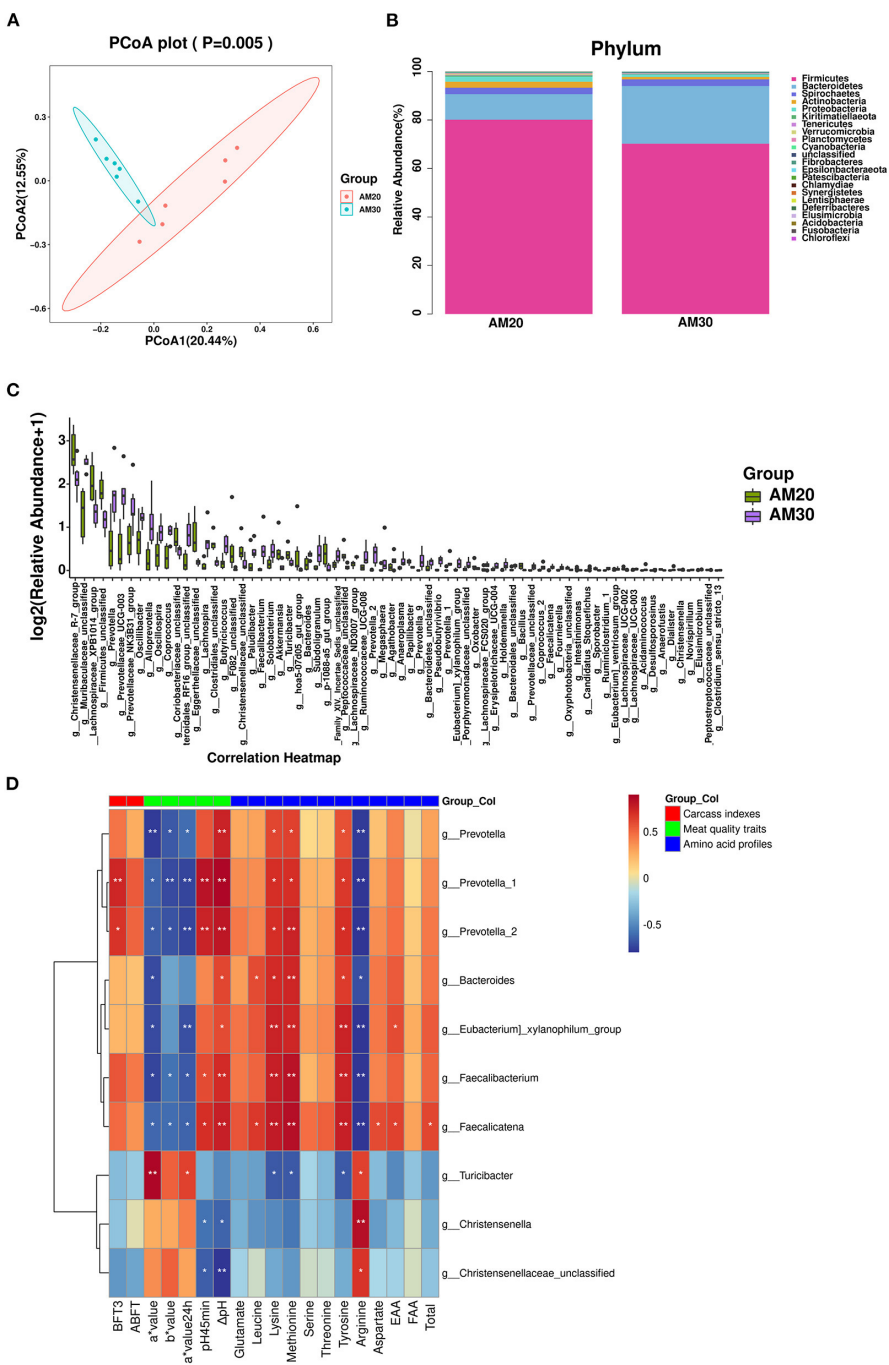
Effects of AM20 and AM30 on gut microbiota in Heigai pigs. **(A)** Unweighted UniFrac PCoA analysis. Each symbol represents a sample. **(B)** Stacked bar chart at phylum level in colonic samples. Different colors indicate different species, different columns represent different subgroups, and the abundance of each subgroup is the average of all biological replicates within that group. **(C)** Significant differences in relative abundance of genera between AM20 and AM30 (Mann-Whitney U test). The box presented the 95% CIs; the line inside denotes the median. **(D)** Spearman's *r* correlation heat map between significantly different gut microbiota and significantly different carcass indexes, meat quality traits, and amino acid profiles for different levels of alfalfa meal treatment. ABFT, average backfat thickness; FAA, flavor amino acids; EAA, essential amino acid. Significance and correlation coefficient was analyzed by Spearman's correlation analysis. *P < 0.05; **P < 0.01.

There were 1,462 common OTUs in the two groups, with means of 5,814 and 4,556 OTUs in AM20 and AM30, respectively. The alpha diversity indices Chao 1 index, Goods coverage, Shannon and Simpson were not significantly different between the two groups (Supplementary Figures 1A–E), indicating that the addition of different levels of alfalfa meal did not affect bacterial richness or evenness. The beta diversity of the bacterial community was analyzed by unweighted PCoA, which indicated a clear distinction between AM30 and AM20 (Figure 2A). At the phylum level (Figure 2B, Supplementary Figure S1F), there were significantly lower relative abundances of *Verrucomicrobia, Planctomycetes*, and *Proteobacteria* in AM30 than in AM20, whereas the opposite patterns were observed for *Bacteroidetes* and *Elusimicrobia*. At the genus level (Figure 2C, Supplementary Figure S1G), there were 70 significantly different genera, including 47 upregulated *Prevotella-2, Faecalicatena*, etc., and 43 downregulated *Akkermansia, Bacillus*, etc., in the gut microbiota of pigs fed AM30 compared to those fed AM20.

### Correlation analysis between significantly different gut microbiota compositions and carcass indices, meat quality traits, fatty acid composition, and amino acid profiles

Among the 70 significantly different genera, 10 genera that were correlated with significantly different carcass indices, meat quality traits, and amino acid profiles were screened out using Spearman correlation analysis (Figure 2D). *Prevotella* is the dominant bacterial genus in the human intestine. Within *Bacteroidetes*, members of the genus *Prevotella* have emerged as underexplored keystone species not only in the human microbiome but also in many animal microbiomes. The results showed that the BFT3 (*P* < 0.01, *P* < 0.05), pH_45min_ (*P* < 0.01), ΔpH (*P* < 0.01), and the contents of lysine (*P* < 0.05), methionine (*P* < 0.05, *P* < 0.01), and tyrosine (*P* < 0.05) were positively correlated with the genera *Prevotella_1* and *Prevotella_2*, whereas the opposite correlations were observed for a^*^ value (*P* < 0.05), b^*^ value (*P* < 0.05) a^*^ value_24h_ (*P* < 0.01), and arginine (*P* < 0.01).

*Faecalicatena fissicatena* was positively correlated with short-chain fatty acid (SCFA) production, since SCFA propionate protects hosts from hypertensive cardiovascular damage and exerts anti-inflammatory effects (16, 17). We found that the genera *Faecalicatena* and *Faecalibacterium* were significantly negatively correlated with a^*^ value (*P* < 0.05), b^*^ value (*P* < 0.05), a^*^ value_24h_ (*P* < 0.05), and content of Arginine (*P* < 0.01), and positively correlated with pH_45min_ (*P* < 0.05), ΔpH (*P* < 0.01), lysine (*P* < 0.01), methionine (*P* < 0.01), and tyrosine (*P* < 0.01) contents. Moreover, *Faecalicatena* was also significantly positively correlated with the contents of lysine (*P* < 0.05), aspartate (*P* < 0.05), EAAs (*P* < 0.05), and total protein (*P* < 0.05).

## Discussion

Recent years have witnessed a dramatic growing interest in pig diets high in fiber, which is of particular significance to the fields of gut health, welfare, and the environment (18, 19), despite its negative impact on nutrient utilization and energy digestibility (20, 21). However, the effects of high alfalfa levels and insoluble dietary fiber on the growth performance, carcass traits, meat qualities, expression of LDM related gene mRNA, and gut microbiota of Chinese indigenous pigs are still unclear. Therefore, the present study compared the effects of 20% and 30% alfalfa meal supplementation on the production performance of Heigai finishing pigs.

In the current study, no significant differences were observed in growth performance between the AM20 and AM30 groups, indicating that Heigai finishing pigs could tolerate the diet with 30% alfalfa meal. It has been suggested that Chinese indigenous pig breeds have better digestibility for crude fiber (3, 22), which is supported by a study that observed no adverse effects on the growth performance of growing-finishing pigs fed a high crude fiber content (20%) from alfalfa (22–27). As a Chinese indigenous pig breed, it's speculated that the Heigai pigs inherited the rough feeding trait and adapt to a higher fiber diet derived from alfalfa meal. Previous studies also suggested that growth performance was not negatively affected by high dietary fiber levels (~20%) from other dietary fiber sources in Chinese indigenous pigs (3, 28). Another possible hypothesis is that diets with 30% alfalfa meal could meet the nutritional needs of Heigai pigs, and thus the pigs had adequate energy to grow.

The lack of a significant difference in carcass weight and dressing percentage after feeding with an elevated level of alfalfa in this study was consistent with previous research (23, 29). In pork production, the excessive deposition of subcutaneous adipose tissue in finishing pigs not only leads to a decreased feed conversion rate but also exerts a negative impact on consumer acceptance of pork. Interestingly, a significantly reduced ABFT was observed in pigs in the AM30 group, implying the improvement of carcass traits to some extent. This finding agreed with numerous previous findings, in which a high-fiber diets supplemented with from alfalfa, bran, and olive cake decreased the BFT in finishing pigs with different genetic backgrounds (13, 29, 30). A series of slow and continuous dietary fiber fermentation processes *in vivo* have been found to maintain blood glucose levels, thus regulating insulin homeostasis in pigs (31–33). These variations in insulin may partly account for the fact that improved energy metabolism and reduced backfat deposition in response to 30% alfalfa in our study.

After slaughter, the relative increase in anaerobic respiration leads a substantial accumulation of lactic acid, resulting in a decrease in the muscle pH value, which exerts an adverse effect on meat quality indices such as tenderness, water-holding capacity, and color. Notably, this study demonstrated that pigs fed the AM30 diet had an effectively decreased pH_45min_ value compared with those fed the AM20 diet. However, the higher level of alfalfa led to a significant decrease in only pH, while drip loss was not significantly affected in this study, which was inconsistent with previous findings regarding the effects of low pH leading to dehydration and contraction of myofibrils (34).

The rate and extent of postmortem pH decline are closely related to glycolysis (35). *PFKM* gene expression was significantly increased, while the *PKM* and *HK2* gene expressions in the LDM was not affected in the pigs of AM30 group. Multiple studies have indicated that the increased *PFKM* expression level in the LDM promotes the biosynthesis of fatty acids in intramuscular adipocytes, which is highly correlated with pork marbling and IMF content (36–38). The result is further supported by histological images and the TG contents of LDM, which suggested increased fatty acid synthesis in the AM30 group.

It has also been purported that high dietary fiber reduces the glycolysis in fresh pork, which may be associated with the improvement in the oxidative fiber composition of muscle (39). A high-fiber diet increased the mRNA abundance of *MyHCI* and decreased *MyHC IIb* and *MyHC IIx* mRNA levels in the LDM of Erhualian pigs, a typical indigenous pig from China. Moreover, the TG content is highly related to the oxidative muscles (40). Consistent with this study, AM30 significantly increased the expression of *MyHCI* and *MyHCIIx* and was accompanied by a decrease in the *MyHCIIb* expression level, providing credible evidence for the difference in meat quality between AM20 and AM30 groups related to the conversion of muscle fibers. The lower pH_45min_ in the AM30 group of pigs may partly account for the lower buffering capacity of type I fiber or differences in the kinetics of postmortem temperature decline of muscles (40). No significant difference in pH_24h_ was observed, which was in line with the breed characteristics of Heigai pigs (41). The proportion of oxidative muscle fibers rich in myoglobin and glycolytic fibers related to pale meat is widely accepted to jointly govern meat color (42), which explains the higher a^*^ values at 45 min and 24 h after slaughter in the AM30 group. The proportion of oxidized muscle fibers and the activity of myoglobin obviously increased with 30% dietary alfalfa inclusion in the current study, thus regulating the pH and meat color of the LDM in Heigai finishing pigs (43, 44).

The fatty acid and amino acid compositions and contents of muscle tissue greatly determine the nutritional quality, meat appearance, and flavor of pork, thus playing an important role in meat quality (45, 46). In this study, the EAAs and total protein content greatly increased without a negative effect on the fatty acid composition in the AM30 group of pigs. These results advocate for the use of AM30 to improve pork nutritional value, which was also supported by a previous study (23).

As the most commonly studied prebiotic (2), fiber can promote hindgut fermentation and microbial growth, yielding energy, and metabolic end products such as volatile fatty acids (VFAs) (47, 48). Volatile fatty acids metabolized in skeletal muscle were found to regulate the energy metabolism of muscle cells by affecting the synthesis and function of mitochondria, which may be a potential mechanism by which dietary fiber interferes with muscle fiber composition and fat content. However, the precise mechanism underlying the regulatory effects of high-level alfalfa on meat quality requires further study due to differences in breeds, nutritional levels, and feeding management.

To further determine whether the alterations mentioned above are attributed to the gut microbiota, the colonic microbial composition of Heigai pigs was analyzed by PCoA. As shown in Figure 2, significant differences were observed in response to dietary alfalfa levels. According to analysis at the phylum level, *Firmicutes* and *Bacteroidetes* were the dominant phyla, exceeding 90% of the relative abundance of the total gut microbiota. Compared with the AM20 group, the relative abundance of *Bacteroidetes* in the AM30 group was significantly increased, while *Firmicutes* abundance was decreased without a significant difference. As the two most abundant bacterial phyla in most mammals (49, 50), the ratio of *Firmicutes*/*Bacteroidetes* validly indicates the status of the intestinal microflora and affects the energy uptake and fat metabolism in the body (51), which was supposed to be related to the decrease in ABFT in pigs in AM30 group. Additionally, the relative abundance of *Prevotella, Prevotella-1*, and *Prevotella-2*, which ferment to produce isovaleric acid, *Christencenella*, a beneficial bacterium in the gut that helps reduce the risk of obesity (52, 53), and *Faecalicatena fissicatena*, which is positively correlated with SCFA production (16, 17), were all significantly higher in the AM30 group. This result suggested that the fatty acids produced by these bacterial fermentations may promote ectopic deposition of IMF and TG content in addition to providing energy requirements.

With the exception of the regulatory role on energy metabolism, the intestinal microbiota possesses specialized enzymes for the utilization of amino acids (54) that mediate the protein metabolism, including digestion, absorption, metabolism, and transformation in the gastrointestinal tract. The proteolytic activity in the large intestine of monogastric animals has been mainly attributed to the genera of *Bacteroides, Propionibacterium, Streptococcus, Fusobacterium, Clostridium*, and *Lactobacillus* (55, 56). Several genera of colonic bacteria are able to produce polyamines in the colonic ecosystem, including *Lactobacillus, Veillonella, Bifidobacterium*, and especially *Bacteroides* (57) of which their relative abundances were significantly higher in AM30. This might explain the increase in threonine, serine, glutamate, methionine, leucine, tyrosine, lysine, aspartate, EAA, FAA, and total amino acid contents in the LDM of the AM30 group. In addition, work in microbe-free and conventional rats has demonstrated the ability of gut microbes to synthesize lysine using ^15^N from ^15^NH4Cl (58). Therefore, we speculated that 30% alfalfa meal improved the amino acid profile of the LDM by upregulating the relative abundance of *Bacteroides* and *Faecalicatena* in the gut microbiota. Additionally, the availability of amino acids from the diet significantly affects both host amino acid metabolism and the utilization of amino acids by the gut microbiota (59), implying that the altering of the amino acid composition in the LDM may result from the bidirectional regulatory relationship between the host and the gut microbiota communities, but further studies need to elucidate the exact mechanism.

## Conclusion

**Figure 3:**
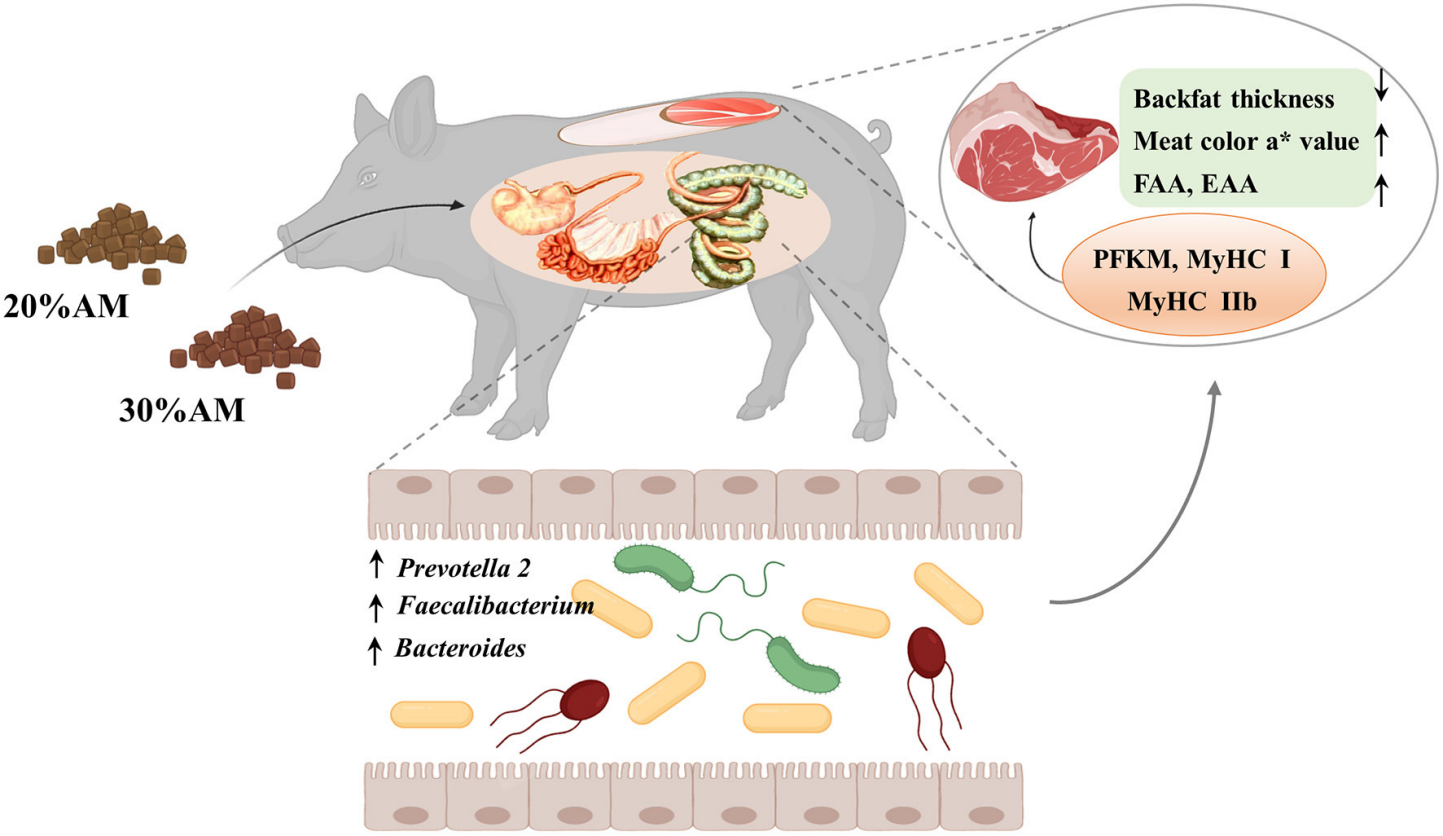
Working model of the effect of alfalfa levels on Heigai pigs.

In summary, the present study demonstrated that a diet supplemented with 30% alfalfa improved carcass traits and meat quality without compromising growth performance, which was possibly associated with the response of the intestinal microbes that played an important role in intervening in the related phenotype and gene expression of Heigai finishing pigs (Figure 3).

## Data availability statement

The data presented in this study are openly available in NCBI at SRA data under reference numbers PRJNA861804.

## Ethics statement

The animal study was reviewed and approved by Zhejiang University Animal Care and Use Committee. Written informed consent was obtained from the owners for the participation of their animals in this study.

## Author contributions

TS and JL designed the research. JL and SZ conducted the research, collected, and assembled the data. JL mainly wrote the manuscript draft. TS, YW, SZ, and XG edited and revised the manuscript. JX and XZ participated in feeding experiment and collected samples. All authors contributed to the article and approved the submitted version.

## Funding

The project was partially supported by the Zaozhuang Talent Program Funding, the Zhejiang Provincial Key R&D Program of China (2021C02008), and the Fundamental Research Funds for the Central University (226-2022-00113) to TS.

## Conflict of interest

JX and XZ are employees of Shandong Chunteng Food Co. Ltd. Zaozhuang, Shandong, China. The remaining authors declare that the research was conducted in the absence of any commercial or financial relationships that could be construed as a potential conflict of interest.

## Publisher's note

All claims expressed in this article are solely those of the authors and do not necessarily represent those of their affiliated organizations, or those of the publisher, the editors and the reviewers. Any product that may be evaluated in this article, or claim that may be made by its manufacturer, is not guaranteed or endorsed by the publisher.

## Abbreviations

a^*^: red-green
ABFT: average backfat thicknesses
ADFI: average daily feed intake
ADG: average daily gain
ASV: amplicon sequence variant
b^*^: yellow-blue
BFT: backfat thicknesses
BW: body weight
EAA: essential amino acid
FAME: fatty acid methyl ester
HPLC: high-performance liquid chromatography
IMF: intramuscular fat
L^*^: lightness
LDM: longissimus dorsi muscle
LSD: least-significant difference
OTU: operational classification unit
PCoA: principal coordinates analysis
SCFA: short-chain fatty acid
TCHO: total cholesterol
TG: triglyceride
VFA: volatile fatty acid.
