# Supplementary material for: Effects of alfalfa levels on carcass traits, meat quality, fatty acid composition, amino acid profile, and gut microflora composition of Heigai pigs
Source: Front Nutr. 2022 Sep 30;9:975455. doi: 10.3389/fnut.2022.975455 (PMC9566568; doi:10.3389/fnut.2022.975455)
Supplement: Supplementary file 1 [file Table_1.DOCX]

**Supplementary Table 1 Ingredient composition and nutritional levels of the diets**

| **Items** | **Diets, %^a^** | |
| --- | --- | --- |
|  | **AM20** | **AM30** |
| Ingredient, % |  |  |
| Corn | 54.4 | 50.4 |
| Soybean meal | 16 | 10 |
| Wheat middlings | 6.4 | 6.4 |
| Compound-premix^b^ | 3.2 | 3.2 |
| Alfalfa meal | 20 | 30 |
| Total | 100 | 100 |
| Energy and Nutrient composition |  |  |
| Metabolic energy (ME kcal/kg) | 2923.72 | 2752.51 |
| Lys, % | 1.03 | 0.82 |
| Met, % | 0.25 | 0.23 |
| Leu, % | 1.40 | 1.28 |
| Arg, % | 0.97 | 0.83 |
| Crude protein (CP %) | 17.01 | 15.89 |
| Crude fiber, % | 6.60 | 8.58 |
| Ca, % | 0.70~0.89 | 0.49~1.01 |

^a^AM20 = 20% alfalfa meal diet; AM30 = 30% alfalfa meal diet.

^b^Provided per kilogram of compound-premix: 0.7~2.1 g Mn from manganese oxide, 1.5~7.5g Fe from iron sulfate, 0.7~2g Zn from zinc sulphate,210~625 mg g Cu from copper sulfate, 5~15 mg I from calcium iodate, and 3~9 mg Se from sodium selenite; 100000~160000 IU vitamin A; 50mg pyridoxine hydrochloride; 50000~120000 IU vitamin D3; 900 mg vitamin E; 40 mg vitamin B1; 50 mg vitamin K; 160 mg riboflavin; 280 mg pantothenic acid;50 mg nicotinamide;30 mg folic acid and 0.85 mg vitamin B12.

**Supplementary Table 2 Sequences used for real-time PCR primers**

| Gene | Primer sequence (5' to 3') | Product size (bp) | GenBank accession no. |
| --- | --- | --- | --- |
| MyHCI^2^ | F^1^: CAAGGCAGAGATGGAGCGGAAG | 152 | NM_213855 |
|  | R^1^: CTCATTGCGGCTGCGTGTCT |  |  |
| MyHCIIa | F: GGAAGCTCGCAACGCAGAAGA | 134 | NM_214136 |
|  | R: TCATCCAGACGGTGCTGTAGGT |  |  |
| MyHCIIb | F: CGGAAGAGGCGGAGGAACAATC | 153 | NM_001104951 |
|  | R: TGACCTGGGACTCGGCAATGT |  |  |
| MyHCIIx | F: GTACAATGCGGTGGGTGCTCTG | 148 | NM_001123141 |
|  | R: GCTGCTGGTTGATGCGAGTGA |  |  |
| PFKM | F: GGCTTTGAGGCTTACACAGG | 85 | XM_021091211.1 |
|  | R: GGATGACCACAAACGGGATG |  |  |
| PKM | F: TCGATGAGATCCTGGAAGCC | 96 | XM_021099125.1 |
|  | R: TCTTCTGAGCCAGGAAGACC |  |  |
| HK2 | F: GTGCCTGGCTAACTTCATGG | 185 | NM_001122987.1 |
|  | R: GGATGGCCTTCCGGATTAGA |  |  |
| GAPDH | F: GTCGGAGTGAACGGATTTGG | 76 | NM_001206359.1 |
|  | R: CAATGTCCACTTTGCCAGAGTTAA |  |  |

^1^F = forward primer; R = reverse primer.

^2^*MyHCI*, *MyHCIIa*, *MyHCIIb*, and *MyHCIIx* were referred in Han et al., (2020)

**Supplementary Table 3 Effects of different levels of alfalfa meal on the fatty acid profiles in** **LDM**

| **Fatty acid (%)^2^** | **Diets^1^** | |
| --- | --- | --- |
|  | **AM20** | **AM30** |
| C14:0 | 3.67±0.52 | 3.42±0.34 |
| C16:0 | 20.00±0.84 | 20.23±0.45 |
| C16:1 | 6.86±0.58 | 6.78±0.47 |
| C18:0 | 17.74±0.83 | 17.18±1.76 |
| C18:1 | 32.97±1.33^b^ | 34.39±1.76^a^ |
| C18:2 | 11.57±3.31 | 10.69±0.39 |
| C18:3 | 0.48±0.07 | 0.74±0.09 |
| C20:0 | 0.76±0.16 | 0.73±0.11 |
| C20:1 | 2.75±0.50 | 2.78±0.44 |
| C20:2 | 0.79±0.13 | 0.81±0.16 |
| C20:4 | 2.40±0.51 | 2.51±0.28 |
| SFA | 42.18±2.01 | 41.56±1.38 |
| UFA | 57.82±2.01 | 58.44±1.38 |
| PUFA | 42.57±1.87 | 43.94±0.99 |
| MUFA | 15.23±3.46 | 14.50±0.7 |

^a,b^ Means in rows with different letters are significantly different (*P*<0.05). Values are mean ± SEM (n = 6).

^1^AM 20 = 20% alfalfa meal diet; AM 30 = 30% alfalfa meal diet.

^2^SFA: saturated fatty acid, SFA =∑ (C14:0, C16:0, C18:0,C20:0); UFA: unsaturated fatty acid, UFA=∑(C16:1,C18:1,C18:2,C18:3,C20:1,C20:2,C20:4); MUFA: monounsaturated fatty acid, MUFA=∑(C16:1,C18:1,C20:1); PUFA: polyunsaturated fatty acid, PUFA=∑(C18:2,C18:3,C20:2,C20:4)

**Supplementary Fig. 1.** OTU Partition and Microbial Diversity Analysis.(A)Venn diagram exhibits the shared and unique operational taxonomic units (OTUs) between two groups. (B) Chao 1 index; (C) Goods coverage; (D) Shannon; (E) Simpson.(F)Significant differences in relative abundance of phyla between AM20 and AM30 (Mann-Whitney U test). The box presented the 95% CIs; the line inside denotes the median. (G) Stacked bar chart at genus level in colonic samples. Different colors indicate different species, different columns represent different subgroups, and the abundance of each subgroup is the average of all biological replicates within that group.
