# Supplementary figures and images for: Effects of alfalfa levels on carcass traits, meat quality, fatty acid composition, amino acid profile, and gut microflora composition of Heigai pigs
Source: Front Nutr. 2022 Sep 30;9:975455. doi: 10.3389/fnut.2022.975455 (PMC9566568; doi:10.3389/fnut.2022.975455)

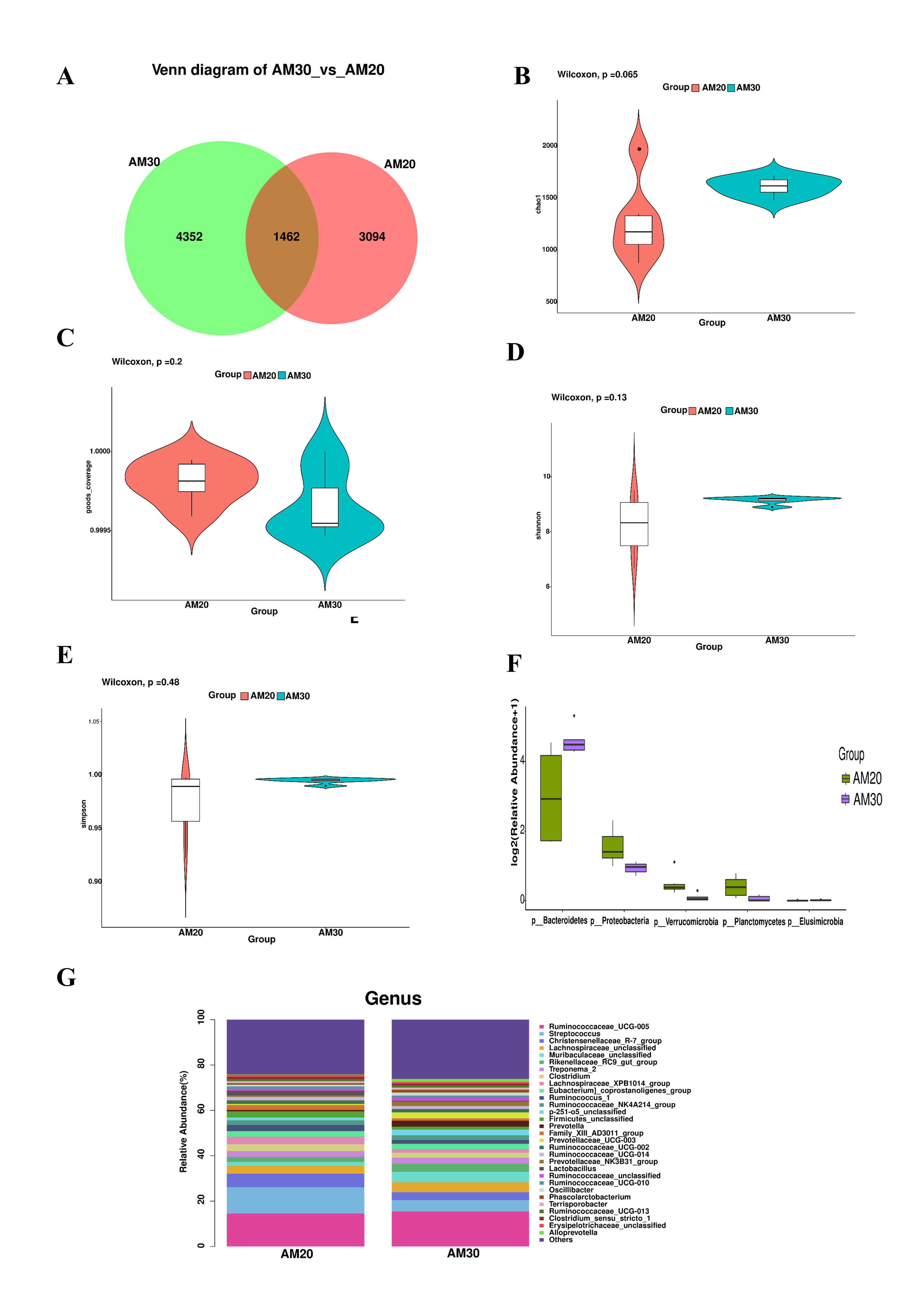

Supplement: Supplementary file 2 [file Image_1.TIF]
